# Successful Management of Peri-Implantitis around Short and Ultrashort Single-Crown Implants: A Case Series with a 3-Year Follow-Up

**DOI:** 10.1155/2019/5302752

**Published:** 2019-09-15

**Authors:** Giorgio Lombardo, Mauro Marincola, Andrea Cicconetti, Miguel Angel Simancas-Pallares, Jacopo Pighi, Jeffrey Lehrberg, Annarita Signoriello, Giovanni Corrocher, Xiomara Serpa-Romero, Luis Armando Vila Sierra, Luisa Arevalo-Tovar, Pier Francesco Nocini

**Affiliations:** ^1^Dentistry and Maxillo-facial Surgery Section, Department of Surgery, Dentistry, Paediatrics and Gynaecology (DIPSCOMI), University of Verona, Piazzale L.A. Scuro 10, 37134 Verona, Italy; ^2^Research Department, Dental Implant Unit, Faculty of Dentistry, University of Cartagena, Cartagena, Colombia; ^3^Department Head and Neck, Oral surgery, University “La Sapienza” Roma, Rome, Italy; ^4^Division of Oral & Craniofacial Health Sciences, Division of Pediatric and Public Health, Adams School of Dentistry, University of North Carolina at Chapel Hill, Chapel Hill, NC 27599, USA; ^5^Department of Biomaterials, Implant Dentistry Centre, Boston, USA; ^6^Research Group in Family Health, University of Magdalena, Santa Marta, Colombia

## Abstract

**Introduction and Aim:**

In case of peri-implantitis, resective surgery is contraindicated for short and ultrashort implants, limiting the treatment options to regenerative surgery or to implant removal. This retrospective case series presents the clinical and radiographic outcomes of a surgical regenerative procedure to treat peri-implantitis around short and ultrashort implants.

**Materials and Methods:**

The study is a retrospective evaluation of patients suffering from peri-implantitis and those who underwent access flap surgery, concomitant chemical and mechanical decontamination of implant surface, and bone grafting using a self-hardening mixture of bone substitutes and biphasic calcium sulfate. No membranes were applied to cover the grafting material, and primary tension-free closure was achieved. The retrospective protocol was reviewed and approved by the Ethics Committee for Clinical Sperimentation (CESC) of Verona and Rovigo, Italy (based in the University of Verona) (Prog. 1863CESC. Date of approval: 2018-07-04).

**Results:**

15 patients (17 implants) have been diagnosed with peri-implantitis after a mean follow-up of 24 months after loading. Implant length was between 5 and 8 mm. 8 patients (10 implants) had a history of periodontitis. At baseline, the mean PD (probing pocket dept) at the deepest site was 8.12 mm, with an average mBI (modified bleeding index) of 2.35 and a mean BD (bone defect depth) of 3.04 mm. At the 3-year follow-up, the CSR was 100%, the mean mBI was 0.88 (average reduction: −1.47), the mean PD was 3.35 mm (mean PD reduction: 4.77 mm), and the mean bone defect was reduced by 1.74 mm, with a mean bone fill of 55%.

**Conclusions:**

The results of the present case series suggest that if accurate surface decontamination is achieved, high survival rate and good clinical and radiographic results can be obtained after 3 years. However, only the histological examination could confirm the growth of new bone in direct contact with the implant surface or if the grafted material only fills the space left by the peri-implant defect.

## 1. Introduction

Several recent studies suggest that short implants (i.e., implants < 8.0 mm in length) perform similarly to longer implants in terms of survival and stability of hard and soft tissues [1–4]. Unfortunately, the prevalence of complications, such as peri-implantitis, is roughly equivalent for short implants and standard-length implants. Peri‐implantitis is a pathological condition occurring in tissues around dental implants, characterized by inflammation in the peri‐implant connective tissue and progressive loss of supporting bone [5].

Resective surgery with implantoplasty [6] is contraindicated for short implants, limiting the treatment options to regenerative surgery or to implant removal.

Regenerative surgery is conditioned by two critical phases, the implant surface decontamination and the defect grafting and/or membrane coverage [7]. The quality and quantity of biofilm attached to the implant surface are significantly influenced by the implant's surface roughness and by implant macrogeometry since rough surfaces tend to accumulate more plaque, and bacterial adhesion starts inside the pits and grooves of the roughened surfaces, wherefrom mechanical removal techniques alone are ineffective [8].

For this reason, in the literature, several chemical topical agents have been proposed in addition to open flap mechanical debridement, but it is hardly possible to compare their adjunctive effect since the grafting protocol differs between one research and another, and there are only few RCTs [9].

Recently, a new topical desiccant (HYBENX Oral Tissue Decontaminant, EPIEN Medical, Inc.) was introduced as an adjunct to ultrasonic and mechanical subgingival debridement in nonsurgical treatment of chronic periodontitis [10, 11] and peri-implantitis [12]. This desiccant is a liquid solution that contains a concentrated blend of sulphonic/sulphuric acids. These acids have a strong affinity to bind to the water present in the biofilm matrix, denaturing the attachment proteins used by bacteria to adhere to the implant surface and allowing more efficient subsequent removal of biofilm microbes and biofilm eradication [13]. To our knowledge, only one case report study using a topical desiccant as an adjunct to air powder abrasives has been published to date [14].

In this paper, we report the three-year results of a regenerative treatment protocol for peri-implantitis that combines the concomitant use of a desiccant agent and an abrasive air powder treatment to decontaminate the implant's surface with the use of a composite graft without the use of membrane.

## 2. Materials and Methods

### 2.1. Study Design and Selection Criteria

In this case series, the study sample was collected at the Clinic of Dental and Maxillofacial Surgery of the University of Verona. Inclusion criteria were as follows: at least one short or ultrashort single-crown plateau-design locking-taper implants presenting PD ≥ 5 mm with BoP+ and/or suppuration at least at one of the six probed sites, presence of semi-circumferential or circumferential peri-implant defects, marginal bone loss ≥2 mm in at least mesial or distal site on radiographic examination, at least one year of loading at the time of diagnosis, absence of implant mobility, and single-crown prosthesis. Exclusion criteria were as follows: cancer of the oral cavity, systemic diseases and/or conditions, radiotherapy of the head/neck in the last 6 months, bisphosphonates use, poor oral hygiene (i.e., FM-VPI > 25%); active periodontal disease (FM-BoP > 25%); and allergy to sulfonates and its derivatives. The retrospective protocol was reviewed and approved by the Ethics Committee for Clinical Sperimentation (CESC) of Verona and Rovigo, Italy (based in the University of Verona) (Prog. 1863CESC. Date of approval: 2018-07-04).

### 2.2. Surgical Protocol

**Figure 1:**
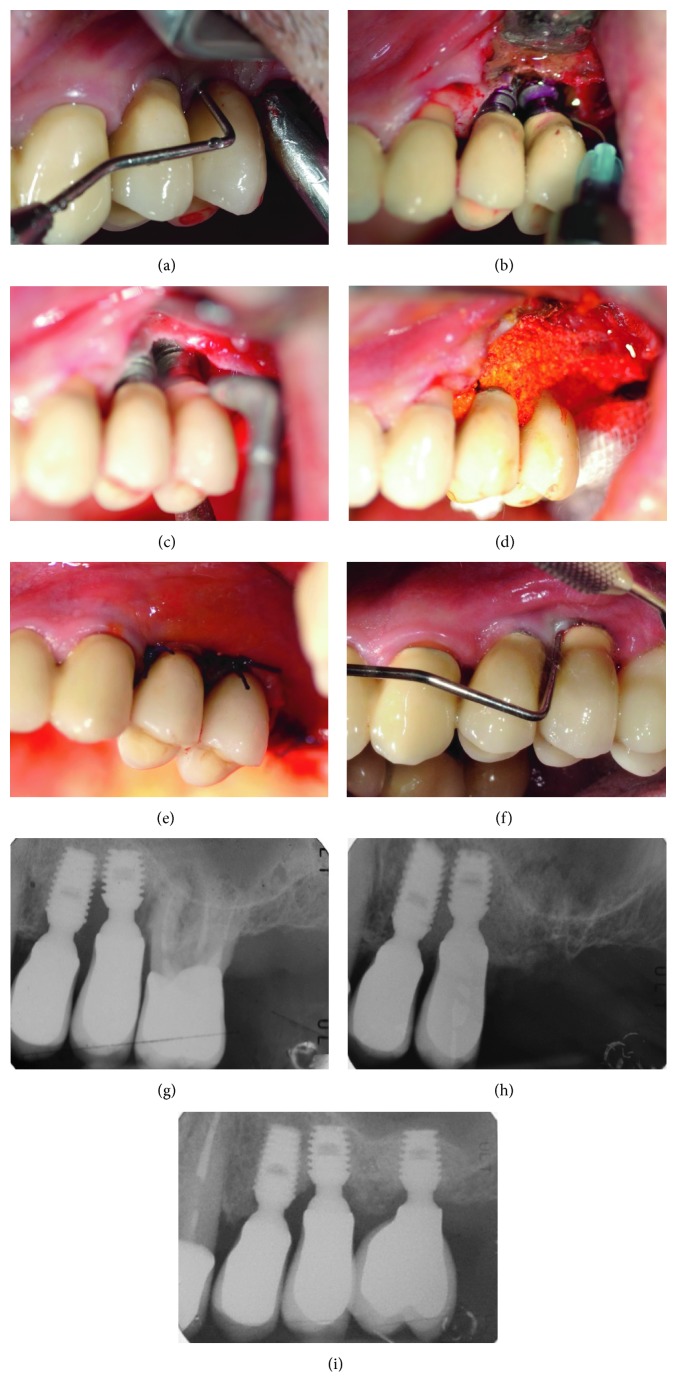
Regenerative treatment of peri-implantitis in the upper posterior jaw in a male patient. (a) Clinical probing before treatment. (b) Application of desiccant. (c) Decontamination with low abrasive air powder. (d) Filling of the peri-implant bone defect. (e) Postoperative clinical aspect. (f) Clinical follow-up 5 years after surgical treatment. (g) Preoperative X-ray. (h) Postoperative X-ray. (i) 5-year follow-up.

**Figure 2:**
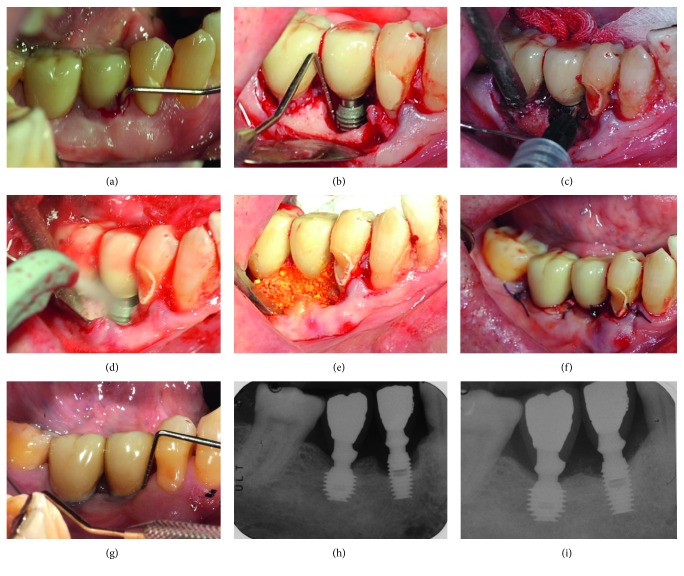
Regenerative treatment of peri-implantitis around an implant placed in the posterior lower jaw of a male patient. (a) Clinical probing before treatment. (b) Intraoperative view of peri-implant bone defect. (c) Application of desiccant. (d) Decontamination with low abrasive air powder. (e) Filling of the peri-implant bone defect. (f) Postoperative clinical aspect. (g) Clinical follow-up 4 years after surgical treatment. (h) Preoperative X-ray. (i) 4-year follow-up.

The surgical procedure is shown in Figures 1to 2, were two patients presenting peri-implantitis were treated using the following protocol. After anesthetizing with a 2% lidocaine solution with 1  mcg/mL epinephrine, the prosthesis was removed and sulcular incisions were made on the buccal and lingual/palatal side in an effort to preserve soft tissue. Full thickness flaps were raised and periosteal incision was performed when a coronal flap advancement was considered appropriate at the time of closure. If present, supracrestal-exposed region of the implant was smoothered using rotating burs.

The surface decontamination procedure consisted of the following 3-step protocol that was repeated twice: (1) application of a desiccant agent (HYBENX Oral Tissue Decontaminant, EPIEN Medical, MN, USA), to the defect and implant surface, with a 60-second incubation period; (2) thorough irrigation of the defect with saline solution to flush out the desiccant; and (3) administration of sodium bicarbonate–based abrasive air powder treatment (Airflow, EMS, Nyon, Switzerland) to all contaminated and exposed parts of the implant surface for 60 seconds.

Bone defects were filled with a composite graft composed of a mixture of 50% of inorganic bone (Bio-Oss, Geistlich Biomaterials) and 50% biphasic calcium sulfate (BondBone, MIS ImplantsTechnologies Ltd.), and Rifampicin was adjuncted (1 vial, Sanofi-Aventis) when considered appropriate.

No membranes were used to coverage the graft, and suture was performed with flap mobilization if needed. The original prosthesis or a temporary healing abutment of adequate dimension was reinserted, to obtain a nonsubmerged primary tension-free healing. Postoperative care included a 0.12% chlorhexidine + 0.05% cetylpyridinium chloride (CPC) rinse (GUM Paroex, Sunstar Suisse S.A.) twice daily for 2 weeks, 1 g of amoxicillin every 12 hours for 7 days, and 800 mg of ibuprofen as needed for pain. The patient was instructed to abstain from brushing for two weeks.

Sutures were removed after two weeks, and patients were placed on an 8- to 12-week recall schedule until the completion of treatment (2 years).

### 2.3. Radiographic Outcomes

**Figure 3:**
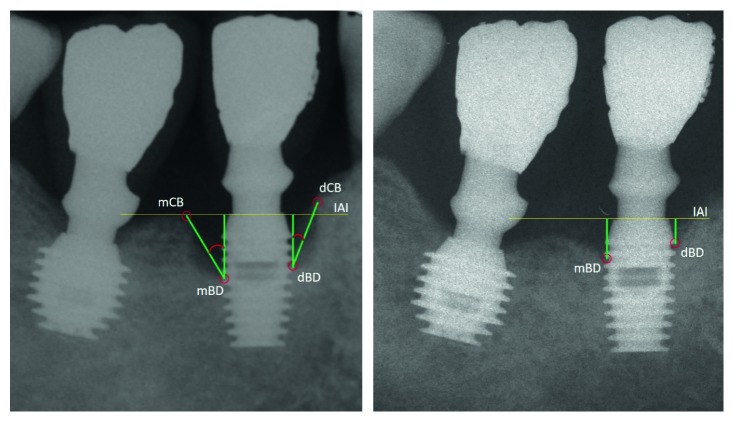
Radiographs were digitalized and used for measurements of bone-level changes between the baseline and last follow-up. mBD: mesial bone defect, dBD: distal bone defect, mCB: mesial crestal bone, dCB: distal crestal bone, IAI: implant-abutment interface.

Changes in the marginal bone level were evaluated using a computerized measurement technique (Rasband, WS, ImageJ, US National Institutes of Health, Bethesda, Maryland, USA), comparing standardized periapical radiographs performed at the time of surgery (peri-implantitis) (T1) and at the follow-up (T2) (Figure 3). Using the implant-abutment interface (IAI) as a reference point, the bottom of the defect (BD) was recorded on mesial and distal sides as the linear distance between the IAI and the more coronal contact point between the bone and the active surface of the implant. The level of the interproximal bone crest (alveolar-crest, AC) was measured as the linear distance between the IAI and the highest point of the proximal bone crest. Finally, the angle of the defect was calculated, between the vertical axis of the implant and the line connecting the fundus of the defect (BD) to the vertex of the interproximal bone peak (AC).

### 2.4. Clinical Outcomes

The stability of the peri-implant tissues was evaluated by means of a millimeter-sized periodontal probe, applying a light intensity force (0.2 N). For each implant site, soft tissue variables examined included the following: probing depth (PD), with the deepest sites categorized as qualifying sites (Q-site); modified bleeding index (mBI); modified plaque index (mPI); recession of the vestibular and palatal/lingual side; and degree of keratinized issue (TK). Peri-implant measurements were performed on six sites. All of the foregoing parameters were recorded immediately before surgery (T1), and at the final follow-up examination (T2).

### 2.5. Statistical Analysis

A database was created using Microsoft Excel, and statistical analysis was conducted using SPSS software (SPSS Inc, IBM). Nominal variables were expressed by frequency distributions, whereas continuous variables were expressed by mean and standard deviation. All variables were expressed in millimeters (mm), except for the FMPS and the FMBS, which were expressed in percentages. A *t*-test for paired samples was used to check the significance of variations in probing depth and radiographic defect depth. Also, *t*-test for impaired samples was used to check the significance of possible differences between implants placed in the upper and lower maxilla at any interval. The minimum level for statistical significance was set at a *p* value of less than 0.05.

## 3. Results

Patient demographics are shown in Table 1. The patient cohort in this study consisted of 15 patients with 17 implants. Eight patients (10 implants) had a history of periodontal disease (53%). The implant population examined in this study consisted of 8 short (i.e., implants 8.0 mm long) and 9 ultrashort (i.e., implants that were ≤6.0 mm long) locking-taper implants. All the implants examined were restored with single ceramic crowns, except one restored with a resin single crown. All surgical procedures and subsequent healing periods occurred without complications, and with minimal postoperative discomfort. The time of the follow-up period, from surgery to the last control examination, was on average 32.1 ± 16.0 months.

The clinical parameters evaluated in this study are summarized in Table 2. The mean PD around implants was significantly reduced by an average of 2.54 ± 1.14 mm, i.e., from a baseline mean value of 5.55 ± 0.84 mm to 3.01 ± 0.73 mm (*p* < 0.0001). The mean number of deep pockets (≥6.0 mm) per implant decreased from 2.41 ± 1.37 to 0.18 ± 0.39 (*p* < 0.0001), and 15.7% of implant sites presented residual pockets (PD ≥ 5.0 mm) at the follow-up examination. Keratinized tissue underwent a minimal average reduction of 0.18 mm, representing an insignificant difference from the baseline to follow-up, with recession occurring for only 0.23 mm.

The average bone filling rate was 57.4% ± 26.5% (60.0% ± 32.5% on mesial side and 53.7% ± 38.7% on distal side). Regions showing the most extensive bone loss at baseline displayed significant bone regeneration at the 3-year follow-up.

At the deepest sites for each implant (Q-sites), the mean PD was significantly reduced by 4.76 ± 1.56 mm, from 8.12 ± 1.58 mm to 3.35 ± 1.17 mm (*p* < 0.0001); mean m-BI of Q-sites decreased from 2.35 ± 0.79 to 0.88 ± 0.93 (*p* < 0.01); and baseline intrabony defect depths at Q-sites revealed an average depth of 3.04 ± 1.14 mm at baseline, which improved to 1.30 ± 0.93 mm at the follow-up (*p* < 0.0001) (Table 2). For Q-sites, the average rate of defect filling was 55.2% ± 31.0%. The baseline mesial angle of the defect (m-Angle) was 22.4° ± 9.3°, and the distal angle of the defect (d-Angle) was 25.2° ± 11.4°. Statistical linear regression failed to demonstrate any dependence of defect reduction from the degree of aperture of the angle.

Table 3 illustrates the outcome variables stratified by location in the upper or lower maxilla. The two groups were comparable at baseline, except for the average probing depth in qualifying sites, which was found significantly higher in implants placed in the mandible. Despite this, the outcome of treatment was quite the same regarding this and all the others variables. Also, there was a trend in finding at baseline a wider keratinized tissue around implant placed in the upper maxilla, even if this difference was not statistically significant and the trend disappeared after treatment.

## 4. Discussion

The use of desiccants in concert with air powder abrasion, followed by composite bone grafting, has been effective in the cases included in this study.

All of the 17 implants treated using the proposed surgical treatment remained in function for the duration of the study. Of the 42 deep pockets (PD ≥ 6 mm) initially discovered at baseline, only 3 remained at follow-up, and average probing pocket depth and bone defect depth were significantly reduced, especially at the level of the deepest sites.

The rationale for the use of the desiccant solution was that it quickly and completely denatures organic molecular biofilm components. This disrupts the molecular attachment mechanisms of the biofilm and enables easier and more effective mechanical debridement procedure [15]. According to this, the topical desiccant was applied before the mechanical debridement.

The use of sulphonic/sulphuric acids was also based on reports demonstrating the clinical effectiveness both on periodontal and peri-implant biofilms. In a split-mouth RCT on patients with chronic periodontitis, nonsurgical therapy with the adjunctive application of desiccant brought to significative greater reduction of BoP than ultrasonic debridement alone after three months [10]. Furthermore, the use of desiccant as monotherapy reached the same bacterial load reduction as UD. Compared with UD, a combined desiccant-UD treatment resulted in a statistically significant greater bacterial load reduction immediately after treatment. Significant better improvements in clinical, microbial, and inflammatory parameters were reported in another split-mouth study on nonsurgical treatment of chronic periodontitis when the application of the desiccant was combined with SRP, rather than SRP alone [11]. In a recently published clinical case series by Pini-Prato et al. [12], the desiccant solution was used to treat sites affected by peri-implant mucositis and peri-implantitis, and a complete resolution of the inflammatory signs was achieved after 3 months without any significant adverse event.

Despite these findings, there is no evidence about the application of this product on the contaminated implant surface during an open flap decontamination procedure, with the exception of a case report involving two implant in a single patient treated with clinical success [14].

Sandblasting systems using different abrasive particles have been used for the surgical treatment of peri-implantitis in animals and humans [16]. A recent literature review reports that decontamination of the implant surface with abrasive powder leads to re-osseointegration in experimental studies on animals. Furthermore, cell proliferation was higher when the surface was decontaminated using the bicarbonate jet rather than using a laser [17]. Based on our knowledge, the present study is the first case series study reporting on implant decontamination procedure with bicarbonate abrasive jet in association with a desiccant antiseptic agent for regenerative treatment of peri-implantitis. For this reason, in this case series study, the reduction of bleeding around implants along with reduction of probing depth is the only one that can support the success of this biofilm decontamination approach.

Current regenerative surgical strategies for the treatment of peri-implantitis could involve the use of barrier membranes to cover the grafted defect [18]; however, the use of membranes has come under criticism because of reports of postsurgical membrane exposure—resulting in increased morbidity and a high prevalence of residual soft tissue inflammation [19].

In a study involving the use of membranes, the prevalence of complications during healing has been shown to be absent in patients treated with bone graft alone, compared with a rate of between 55% and 60% in patients treated with a membrane [20]. And, in another study, the prevalence of membrane exposure was found to be as high as 90.2%, with a peak between the second (43.8%) and the seventh (34.4%) week of healing [21].

Several controlled clinical trials compared the results of procedures performed in the presence or absence of barrier membranes [19–22], the results of which did not report significantly improved outcomes in terms of implant prognosis for procedures using membranes [22].

Because of extensive heterogeneity of variables like decontamination protocol, grafting material, and systemic/local antimicrobics, it is difficult to compare the results of different studies. Moreover, only histology can confirm whether the graft has made direct contact with the implant surface or if it is acting as a filler only. In lack of histological analysis, the treatment's efficacy can only be supported by similar protocols reported in the literature. For example, Benheke et al. [23] treated peri-implantitis using a corticocancellous bone graft without membrane coverage, of which there were only two reported failures over 25 implants treated: one due to graft mobility (occurring 30 days after surgery) and the other due to graft resorption. After 3 years, the clinical and radiographic outcomes accord with the outcomes reported here, although no flap dehiscence/resorption was detected during healing, maybe due to the choice of using only particulate bone graft instead of blocks.

Roccuzzo et al. examined the use of deproteinized bovine bone mineral with 10% collagen and without membrane coverage or submerged healing in patients suffering from peri-implantitis [24]. After 7 years, only 4 out of 26 implants failed and 4 needed additional antibiotics or additional surgery during supportive periodontal therapy (SPT) to survive [24].

Short and ultrashort locking-taper implants are mainly positioned 2-mm subcrestally and in posterior atrophic areas, where the alveolar ridges are usually wide and the defects circumferential and contentive.

In the present study, all the patients enrolled presented with circumferential contentive defects (Class Ie) or with semicircular bone resorption associated with loss of the vestibular/palatal bone (Class Ib) [25]. In addition, in this study, we used as a grafting material a mixture of inorganic bovine bone material and biphasic calcium sulfate, where the inorganic bovine bone acts as a long-term space maintainer and the biphasic calcium sulfate acts as short-range space maintainer, which completely degrades in strict relation to the bone formation rate (4–10 weeks), at the same time conferring to the composite bone graft the ability to harden and to remain in place even in the presence of blood and saliva.

On this basis, although in some other circumstances the use of membranes may be considered beneficial (e.g., specific noncontentive bone defects) [26], in the present study to allow a minimal invasive approach, graft-retaining membranes were not used.

The importance of optimal plaque control, both before and after surgical therapy, has been extensively described [27–30]. Both FM-VPS and FM-Bop scores were on average very low at the follow-up; however, even if the percentage of bleeding sites decreased from 75% to 29%, only 47% of the qualifying sites (8/17) were found to have ceased bleeding at the follow-up. In agreement with a recent study examining a regenerative approach around implants and reporting the ceasing of bleeding at probing in only 49.3% of sites (37/75) after 1 year [31], the results of our study seem to underline the fact that a complete healing of inflamed peri-implant tissues is not an easily achievable goal.

This is particularly true for patients with a history of periodontitis. In this study, patients underwent a personalized supportive periodontal treatment protocol (SPT), the content of which was influenced by the presence of periodontitis. In fact, patients with an history of periodontitis had a frequency of recall for SPT that was on average 4.5 months, whereas healthy patients had an average frequency of oral hygiene recalls of 6.9 months. Nevertheless, periodontal patients were found to have a higher percentage of bleeding (17%) than nonperiodontal patients (5%), and this justifies the fact that such patients should follow a SPT closer in time to have a long-term success of the therapy.

## 5. Conclusion

With the limitations of this retrospective case series, the protocol proposed in this study for regenerative treatment of peri-implantitis kept in function all the implants of the sample. The grafting technique proved to be effective from a radiographic point of view. The kind of bone defect treated, mainly circumferential, allowed to avoid the use of barrier membranes in order to reduce patient morbidity. The sample consisted of short and ultrashort implants, and a possible deduction, which requires further clinical confirmations from randomized prospective studies, is that the length of the implant does not affect treatment possibilities if the implant stability is preserved. Reduction of inflammation and clinical and radiographic improvements encourage further and more accurate research on the proposed decontamination method.

## Figures and Tables

**Table 1:** Demographic variables.

	Patients	Implants
N	15	17
Gender (M/F)	10/5	11/6
ASA status (I/II)	8/7	11/6
Length (8-mm length/≤6.0-mm length implants)	9/8	8/9
Jaw (upper/lower)	7/8	8/9
Periodontal history (Y/N)	8/7	10/7
Smoking (Y/N)	5/10	6/11

**Table 2:** Clinical parameters of the implant population. PD: probing depth; REC: recession; m-BI: modified bleeding index; mPLI: modified plaque index; av-BD: average bone defect depth; m-BD: mesial bone defect depth; d-BD: distal bone defect depth; m-Angle: mesial angle of defect; d-Angle: distal angle of defect; KT: keratinized tissue; FM-BoP: full-mouth bleeding on probing; FM-VPI: full-mouth visible plaque index; Q-sites: deepest site; deep pockets: PD ≥ 6.0 mm.

	Baseline	Follow-up	Variation	*p*
FM-BoP	—	12.24% ± 11.6%	—	—
FM-VPI	—	8.41% ± 9.79%	—	—
PD	5.55 ± 0.84	3.01 ± 0.74	−2.54 ± 1.14	<0.0001
REC	0.35 ± 0.61	0.58 ± 1.23	−0.23 ± 0.83	Ns
Keratinized tissue (KT)	1.00 ± 1.27	0.82 ± 1.18	−0.18 ± 0.39	Ns
mBI	1.27 ± 0.56	0.65 ± 0.70	−0.63 ± 1.07	<0.05
mPI	0.18 ± 0.35	0.15 ± 0.34	−0.03 ± 0.51	Ns
Number of deep pockets per implant	2.41 ± 1.37	0.18 ± 0.39	2.23 ± 1.52	<0.0001
PD-Q-sites	8.12 ± 1.58	3.35 ± 1.17	−4.76 ± 1.56	<0.0001
mBI-Q-sites	2.35 ± 0.79	0.88 ± 0.93	−1.71 ± 1.05	<0.01
mPI-Q-sites	0.23 ± 0.66	0.18 ± 0.53	−0.06 ± 0.24	Ns
m-angle	22.42 ± 9.26	—	—	—
d-angle	25.26 ± 11.38	—	—	—
m-BD	2.57 ± 1.15	1.03 ± 0.82	−1.54 ± 0.94	<0.0001
d-BD	2.97 ± 1.32	1.26 ± 0.98	−1.70 ± 1.41	<0.001
BD Q-sites	3.04 ± 1.14	1.30 ± 0.93	−1.74 ± 1.19	<0.0001

**Table 3:** Clinical parameters of the implants population stratified by maxilla or mandible placement. Parameters evaluated include probing depth (PD); bleeding index (mPLI); plaque index (mBI); mesial bone defect (m-Bone defect); mesial angle of defect (m-Angle); distal angle of defect (d-Angle); keratinized tissue (KT); full-mouth BoP (FM-BoP); full-mouth visible plaque index (FM-VPI).

	Baseline		*p*	Follow-up		*p*	Variation		*p*
	Lower	Upper		Lower	Upper		Lower	Upper	
PD	5.88 ± 0.45	5.16 ± 1.03	0.07	2.92 ± 0.74	3.10 ± 0.78	0.67	2.96 ± 0.93	2.06 ± 1.23	0.09
mBI	1.39 ± 0.60	1.15 ± 0.53	0.37	0.68 ± 0.74	0.60 ± 0.69	0.81	0.70 ± 1.25	0.54 ± 0.90	0.54
PI	0.17 ± 0.35	0.19 ± 0.37	1	0.17 ± 0.35	0.13 ± 0.35	0.81	0.00 ± 0.50	0.06 ± 0.56	0.74
Rec	0.11 ± 0.33	0.62 ± 0.74	0.17	0.22 ± 0.44	1.00 ± 1.69	0.32	−0.11 ± 0.60	−0.37 ± 1.06	0.96
KT	0.44 ± 1.01	1.62 ± 1.30	0.06	0.33 ± 0.70	1.37 ± 1.40	0.11	0.11 ± 0.33	0.25 ± 0.46	0.67
FM-BoP	—	—	—	14 ± 15%	10.2 ± 6.5%	0.96	—	—	—
FM-VPI	—	—	—	5.1 ± 5.9%	12.1 ± 12.2%	0.24	—	—	—
PD ≥ 6.0 mm	2.67 ± 1.50	2.12 ± 1.25	0.64	0.00 ± 0.00	0.37 ± 0.52	0.08	2.67 ± 1.50	1.75 ± 1.49	0.64
PD Q-sites	8.89 ± 1.36	7.25 ± 1.39	0.04^*∗*^	3.44 ± 1.23	3.25 ± 1.16	0.81	5.44 ± 1.24	4.00 ± 1.60	0.09
mBI Q-sites	2.44 ± 0.88	2.25 ± 0.71	0.54	0.67 ± 0.87	1.12 ± 0.99	0.37	2.00 ± 1.11	1.37 ± 0.92	0.24
mPI Q-sites	0.22 ± 0.67	0.25 ± 0.71	1	0.11 ± 0.33	0.25 ± 0.71	1	0.11 ± 0.33	0.00 ± 0.00	0.74
Mesial angle	22.29 ± 8.23	22.58 ± 10.90	1	—	—	—	—	—	—
Distal angle	28.06 ± 11.9	22.08 ± 10.60	0.28	—	—	—	—	—	—
m-BD	2.91 ± 1.33	2.18 ± 0.82	0.20	1.37 ± 0.81	0.64 ± 0.66	0.06	1.54 ± 1.02	1.54 ± 0.89	0.74
d-BD	3.06 ± 1.49	2.86 ± 1.21	0.67	1.22 ± 0.90	1.31 ± 1.12	0.89	1.84 ± 1.39	1.55 ± 1.52	0.61
BD Q-sites	3.17 ± 1.13	2.89 ± 1.21	0.54	1.41 ± 0.94	1.17 ± 0.99	0.48	1.76 ± 1.13	1.33 ± 0.47	1

## Data Availability

The data used to support the findings of this study are available from the corresponding author upon request.
